# Effect of Hydrophobic Polypeptide Length on Performances of Thermo-Sensitive Hydrogels

**DOI:** 10.3390/molecules23051017

**Published:** 2018-04-26

**Authors:** Jiandong Han, Xingyu Zhao, Weiguo Xu, Wei Wang, Yuping Han, Xiangru Feng

**Affiliations:** 1 Department of Chemistry, Changchun University of Science and Technology, Changchun 130022, China; jdhan@ciac.ac.cn; 2 Key Laboratory of Polymer Ecomaterials, Changchun Institute of Applied Chemistry, Chinese Academy of Sciences, Changchun 130022, China; star20012002@163.com (X.Z.); wgxu@ciac.ac.cn (W.X.); 3 Department of Urology, China-Japan Union Hospital of Jilin University, Changchun 130033, China

**Keywords:** amphiphilicity, phase change, polyamino acids, degradability

## Abstract

Thermosensitive gels are commonly used as drug carriers in medical fields, mainly due to their convenient processing and easy functionalization. However, their overall performance has been severely affected by their unsatisfying biocompatibility and biodegradability. To this end, we synthesized poly(l-alanine) (PLAla)-based thermosensitive hydrogels with different degrees of polymerization by ring-opening polymerization. The obtained mPEG_45_−PLAla copolymers showed distinct transition temperatures and degradation abilities. It was found that slight changes in the length of hydrophobic side groups had a decisive effect on the gelation behavior of the polypeptide hydrogel. Longer hydrophobic ends led to a lower gelation temperature of gel at the same concentration, which implied better gelation capability. The hydrogels showed rapid gelling, enhanced biocompatibility, and better degradability. Therefore, this thermosensitive hydrogel is a promising material for biomedical application.

## 1. Introduction

The application of hydrogels in the biomedical field has rapidly increased over the past decade, including use in three-dimensional (3D) cell culture [1,2,3], drug delivery [4,5,6], and tissue engineering [7,8,9]. Polymer hydrogels can trap a large amount of moisture inside for easier cell membrane penetration and drug transmission. They enjoy excellent physical properties and exhibit a controllable degradation process. On account of the incomparable convenience, the in situ gelation of biodegradable hydrogels has aroused great interest in many researchers. Among numerous biodegradable hydrogels, thermosensitive hydrogels have specific advantages. On the one hand, they are rather safe for clinical utilization because the common thermosensitive gel preparation process does not involve the use of organic solvents. On the other hand, the gelling conditions of heat-sensitive hydrogels are easy to control, making them applicable in the field of biomedicine [10,11,12].

Thermosensitive hydrogels can be generated from block copolymers consisting of hydrophilic poly(ethylene glycol) (PEG) and hydrophobic moieties such as poly(lactic acid) (PLA) [13,14], poly(lactic-*co*-glycolic acid) (PLGA) [15,16,17], poly(ε-caprolactone) (PCL) [18,19,20], polyamidoamine (PAMAM) [21], and so on. Thermosensitive hydrogels are soluble at low temperatures, which facilitates the introduction of chemotherapeutic drugs [22,23,24], functional proteins [25], and cells. Due to the amorphous form of the hydrogel at low temperatures, it can be seamlessly filled into injury sites [26,27]. At body temperature, the soluble hydrogels turn into a gel state and provide a depot for the encapsulated medical agents to achieve sustained release and long-term therapeutic effect [28]. For example, growth factors can be slowly and continuously released locally for the efficient repair of bone and nerve tissues [29,30,31]. The hydrogels can also function as 3D scaffolds and as a nutrient resource for cell growth in tissue engineering [32], aiming to help cells grow in a more uniform manner and promote cell proliferation [33].

In 2012, Qian’s team prepared a bionic hydrogel composed of three components (i.e., triblock PEG−PCL−PEG copolymer (PECE), nano-hydroxyapatite (n-HA), and collagen), which showed satisfactory efficacy in skull repair [34]. In 2015, Chen’s team engineered a thermosensitive PLGA−PEG−PLGA hydrogel loaded with 5-fluorouracil for the prevention of postoperative tendon adhesion, and acquired a good histological score [35]. However, despite the wide application of thermosensitive hydrogels in various diseases in clinical practice, they are not free from flaws. Some hydrogels are difficult to gelatinize, and the degradation time is uncontrollable. Moreover, hydrogels with poor biocompatibility always lead to severe tissue damages. Therefore, there is an urgent need to develop thermosensitive polypeptide polymers with good biocompatibility and biodegradability to avoid the above problems.

Towards this aim, peptide systems have been developed to avoid evoking the formation of an acidic microenvironment during degradation, and thus reduce the damages to surrounding tissues and minimize the adverse effects toward the bioactivity of the loaded protein or cells. Apart from this, the gelation performance of the polypeptide copolymer can be tuned by copolymerization with various hydrophobic or hydrophilic amino acid monomers, which widens the clinical applications. In 2011, Jeong’s team reported the synthesis of poly(alanine-*co*-leucine)−poloxamer−poly(alanine-*co*-leucine) (PAL−PLX−PAL) hydrogels, which exhibited a sustained drug release pattern without producing evident inflammatory effects [22]. In 2017, methoxy poly(ethylene glycol)-*block*-poly(l-alanine-*co*-l-phenylalanine) mPEG-*b*-PLAF hydrogel was synthesized by the ring-opening polymerization (ROP) reaction of l-alanine *N*-carboxyanhydrides (l-Ala NCA) and l-phenylalanine NCA, with amino-terminated mPEG (mPEG-NH_2_) as a macroinitiator. Encapsulated with combretastatin A4 (CA4) and doxorubicin (DOX), mPEG−*b*−PLAF hydrogel had an excellent inhibitory effect on the proliferation of B16F10 melanoma cells [36].

In this work, mPEG−poly(l-alanine) (mPEG−PLAla) polymers with different degrees of polymerization (DPs) were synthesized and named mPEG_45_−PLAla_30_, mPEG_45_−PLAla_22_, and mPEG_45_−PLAla_14_. The influence of different DPs on the gelation properties of hydrogels was studied. It was found that hydrogels with higher DP showed better gelation ability than those with lower DP. In addition, the gelation behavior, tissue safety, and cytotoxicity of these polymers in vitro and in vivo were also tested and evaluated. The study revealed that these hydrogels had notable potential for employment in the biomedical field.

## 2. Results and Discussion

### 2.1. Material Synthesis and Structure Characterization

**Scheme 1:**
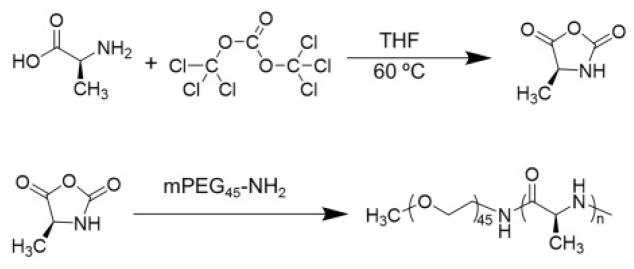
Synthetic routes of l-alanine *N*-carboxyanhydrides (l-Ala NCA) and methoxy poly(ethylene glycol)−poly(l-alanine) (mPEG_45_−PLAla).

Triphosgene and l-alanine were synthesized into l-Ala NCA in dried tetrahydrofuran (THF) solvent, as shown in Scheme 1. The polypeptide hydrogels were produced via the ROP of l-Ala NCA initiated by mPEG_45_-NH_2_. The mPEG_45_−PLAla copolymers with different DPs were synthesized by altering the feed amount. The DPs of PLAla were determined by contrasting the integral of the methyl peaks of side chains (−C*H*_3_) with the methylene peak of PEG (−C*H*_2_C*H*_2_O−). The DPs of mPEG_45_−PLAla_30_, mPEG_45_−PLAla_22_, and mPEG_45_−PLAla_14_ were 30, 22, and 14, respectively.

**Figure 1:**
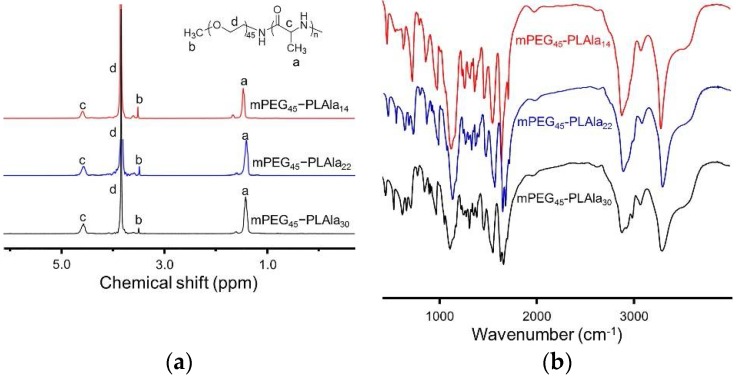
Structure characterization of mPEG_45_−PLAla. (**a**) ^1^H-NMR spectra of mPEG_45_−PLAla_30_, mPEG_45_−PLAla_22_, and mPEG_45_−PLAla_14_, where the subscripts indicate the degree of polymerization (DP); (**b**) Fourier-transform infrared (FT-IR) spectra of mPEG_45_−PLAla_30_, mPEG_45_−PLAla_22_, and mPEG_45_−PLAla_14_.

The typical proton nuclear magnetic resonance (^1^H-NMR) spectrum of block copolymer is shown in Figure 1a, and all peaks could be accurately assigned. The peaks at 1.47, 3.40, 3.50–3.80, and 4.64 ppm stood for the protons of alanine methyl, mPEG terminal methoxy, mPEG backbone, and polymer methine, respectively, indicating the successful synthesis of the three polymers.

The secondary structure of the copolymer was studied by FT-IR. In Figure 1b, characteristic peaks of amide bond at 1627 cm^−1^ and 1544 cm^−1^ were observed, indicating that all copolymers went through the main β-sheet conformation.

### 2.2. Gelation Ability and Internal Appearance

**Figure 2:**
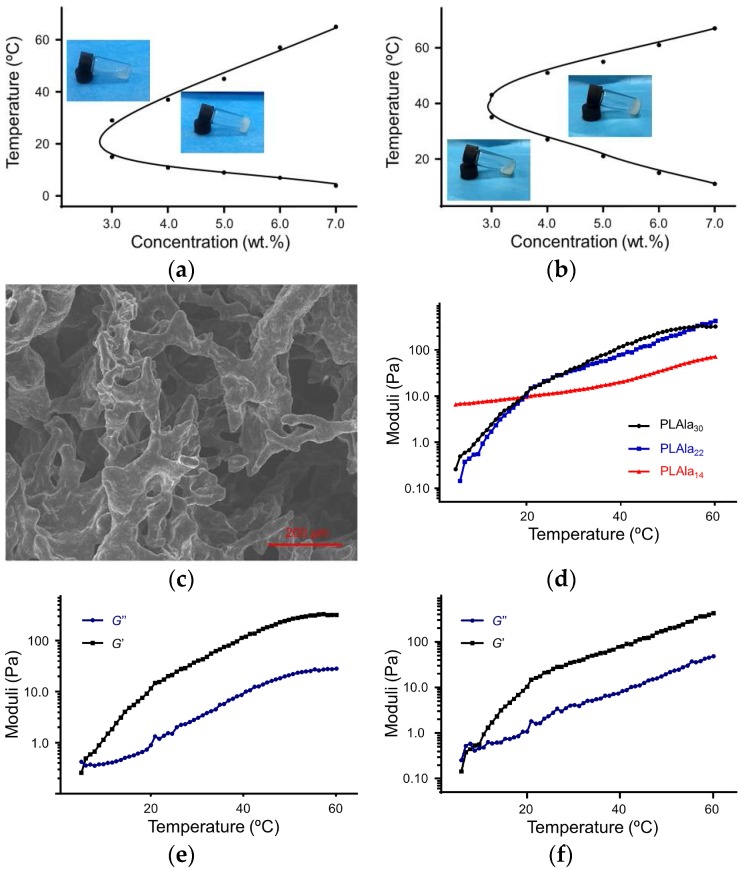
Gelatinization characteristics of mPEG_45_−PLAla. (**a**) Solution–gel phase diagrams of the mPEG_45_−PLAla_30_ and (**b**) mPEG_45_−PLAla_22_ copolymer solutions; (**c**) Scanning electron microscope (SEM) image of mPEG_45_−PLAla_30_ hydrogels formed at 40 °C; (**d**) *G*’ of the mPEG_45_−PLAla_30_ (PLAla_30_), mPEG_45_−PLAla_22_ (PLAla_22_), and mPEG_45_−PLAla_14_ (PLAla_14_) at the concentration of 5.0 wt. % in phosphate-buffered saline (PBS) solution; (**e**) Changes of *G*’ and *G*” of mPEG_45_−PLAla_30_ and (**f**) mPEG_45_−PLAla_22_ in PBS solutions (5 wt. %).

The tube inversion method was used to determine the transition temperatures. Polypeptides were solubilized in a phosphate-buffered saline (PBS) solution, and went through a solution–gel transition with the change in temperature. The samples were defined as gel if they stayed still when the vial was inverted for as long as 30 s. The phase diagrams of mPEG_45_−PLAla_30_ and mPEG_45_−PLAla_22_ copolymers are shown in Figure 2a,b. Only copolymers with a DP of no less than 22 could undergo solution–gel transition at the concentration of 3.0–8.0 wt. %. It was noticeable that mPEG_45_−PLAla_22_ showed a higher gel transition temperature than mPEG_45_−PLAla_30_. The critical gelation temperatures (CGTs) of mPEG_45_−PLAla_30_ and mPEG_45_−PLAla_22_ were 5 °C and 15 °C, respectively, at the concentration of 5.0 wt. %. The lower CGT of mPEG_45_−PLAla_30_ could be explained by longer hydrophobic end than that of mPEG_45_−PLAla_22_ [37].

Solution phase behaviors of the three copolymers at different temperatures were investigated. The images in Figure 2a,b showed the mPEG_45_−PLAla_30_ and mPEG_45_−PLAla_22_ solutions in PBS (5.0 wt. %) at 4 °C and 37 °C. Although all copolymers were dispersed in PBS at 4 °C, the mPEG_45_−PLAla_14_ formed a clear solution while mPEG_45_−PLAla_30_ and mPEG_45_−PLAla_22_ showed a turbid state. When temperature rose to 37 °C, mPEG_45_−PLAla_30_ and mPEG_45_−PLAla_22_ were observed to be stably gelled within 5 min, while mPEG_45_−PLala_14_ remained in a viscous flow state. This was because mPEG_45_−PLala_14_ had better water solubility. Further, the morphology of the mPEG_45_−PLAla_30_ hydrogel (5.0 wt. %) was observed by scanning electron microscope (SEM). As can be seen in Figure 2c, it was found that there were numerous tiny pores in the mPEG_45_−PLAla_30_ gel, which was due to the 3D network structure of the gel. These pores showed a size of about 100 μm and were evenly distributed within the hydrogel, making them suitable as a depot for therapeutic agents. These findings prove that mPEG_45_−PLAla_30_ and mPEG_45_−PLAla_22_ hydrogels feature good stability at body temperature and are suitable for biomedical applications.

### 2.3. Mechanical Performance Test

Thermally induced storage modulus (*G*′) and loss modulus (*G*″) changes of the three copolymers were obtained by dynamic mechanical analysis. *G*′ represents the systematic gel-like behavior of the elastic component of the complex modulus, and *G*″ is an index of the viscous component of the complex modulus and a measure of the sol-like behavior. The intersection of *G*′ and *G*″ reflects the sol–gel transition. As shown in Figure 2d, the *G*′ of mPEG_45_−PLAla_30_ and mPEG_45_−PLAla_22_ obviously increased along with rising temperatures. However, only a minor increment in *G*′ was seen in mPEG_45_−PLAla_14_, and no significant change in *G*′ was detected, even when the temperature rose to about 50 °C, implying that there was no sol–gel modification of mPEG_45_−PLAla_14_ within the experimental temperature range. The reason was that hydrophilic mPEG was longer than the hydrophobic end made up of polypeptides. Therefore, with a longer hydrophilic segment, mPEG_45_−PLAla_14_ showed better water solubility than mPEG_45_−PLAla_30_ and mPEG_45_−PLAla_22_, thus featuring less obvious sol–gel change. Notably, the length of the hydrophobic side chain had a remarkable effect on the gelation function of the mPEG−peptide block copolymer. 

In Figure 2e,f, *G*′ was smaller than *G*″ when the temperature was lower than CGTs (5 °C in mPEG_45_−PLAla_30_ and 12 °C in mPEG_45_−PLAla_22_), which reflected the viscous state of hydrogels. As the temperature increased above CGTs, *G*′ was sharply elevated and surpassed *G*″, confirming the gel formation. This result was in accordance with the findings in Figure 2a,b.

### 2.4. Mechanism of Gelatinization

**Figure 3:**
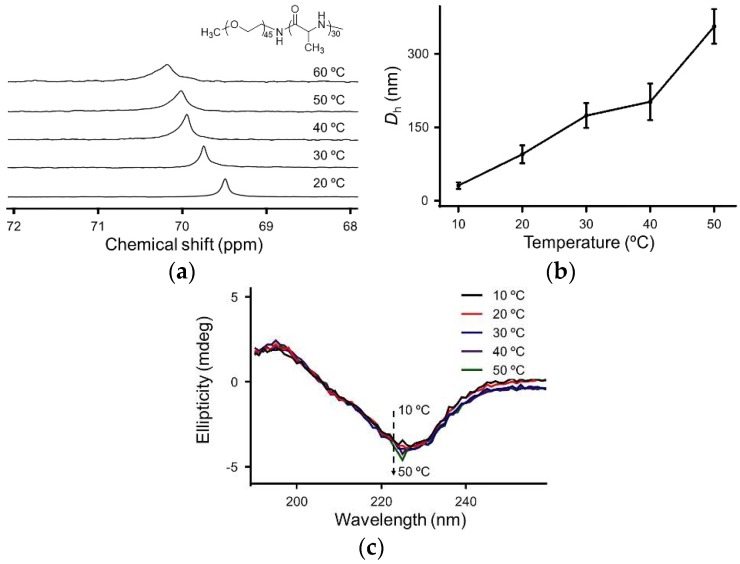
Gelation mechanism of the hydrogels. (**a**) ^13^C-NMR spectra (in D_2_O) of 5.0 wt. % mPEG_45_−PLAla_30_ solution as a function of temperature; (**b**) Average hydrodynamic diameter *(D_h_)* of micelles of mPEG_45_−PLAla_30_ as a function of temperature in water (5.0 μg mL^−1^); (**c**) Circular dichroism (CD) spectra of mPEG_45_−PLAla_30_ (0.05 mg mL^−1^) in aqueous solution as a function of temperature.

In order to study the mechanism of sol–gel transition, the nuclear magnetic peak changes, diameter changes, and conformation evolution of polypeptides in response to temperature were tested by carbon nuclear magnetic resonance (^13^C-NMR), dynamic light scattering (DLS), and circular dichroism (CD). As shown in Figure 3a, when temperature rose from 20 to 60 °C, the characteristic peak of PEG gradually moved from 69.7 to 70.3 ppm, which revealed continuous dehydration of the PEG block during the heat-induced sol–gel transition process. Two main reasons accounted for the gelation of polymers. First, the interaction between hydrophobic blocks became stronger as temperature increased. Secondly, dehydration of PEG segments facilitated the aggregation of the hydrogels [38].

As seen in Figure 3b, the diameter changes of mPEG_45_−PLAla_30_ were determined at the concentration of 5.0 μg mL^−1^. At 10 °C, the average hydrodynamic diameter (*D*_h_) of mPEG_45_−PLAla_30_ was about 31.4 nm. When the temperature rose to 20 °C and 50 °C, the number swiftly increased to 95.9 nm and 356.2 nm, respectively. The dramatic changes in particle size could be explained by the interaction between the shell of PEG and core of polypeptides caused by the dehydration processof PEG [38].

In Figure 3c, the CD spectrum illustrated the alteration of the secondary structure of the aqueous mPEG_45_−PLAla_30_ during the sol–gel transition process. The two typical bands corresponding to the β-sheet conformation, a positive Cotton band at 195 nm and a negative Cotton band at 226 nm, were clearly shown. The above results revealed that the synthesized thermosensitive hydrogel had good physicochemical properties and a clear solution−gel transformation mechanism.

### 2.5. Degradability Test and Pathological Analysis

**Figure 4:**
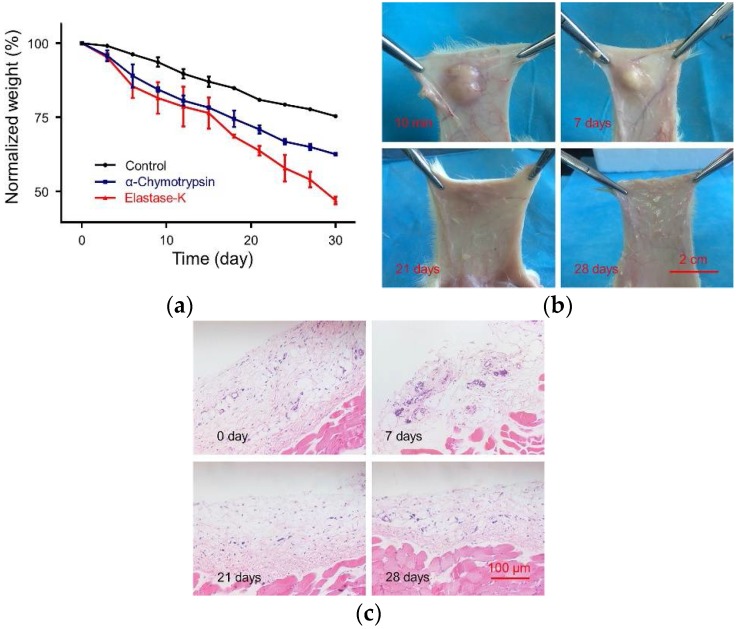
Degradation of hydrogel in vitro and in vivo, and histological analysis of skin. (**a**) Mass loss curves of in vitro degradation of hydrogels in PBS, and PBS with elastase-K or α-chymotrypsin (0.2 mg mL^−1^) groups; (**b**) Images of in vivo gel maintenance at 10 min, 7, 14, 21, and 28 days after the injection of 5.0 wt. % mPEG_45_−PLAla_30_ hydrogels; (**c**) Hematoxylin and eosin (H&E) images of the skin tissue near the hydrogels on day 7, 14, 21, and 28, respectively.

Since hydrogels are often used in the biomedical field, biodegradability is a decisive factor for clinical application. If the degradation rate of the hydrogel is too high, it will lead to a rapid release of the payload. In contrast, if the hydrogel requires a long time to degrade, then it will probably remain at the injection site even after all drugs are released, which is inconvenient for further treatment. The in vitro degradation of mPEG_45_−PLAla_30_ hydrogel (5.0 wt. %) was evaluated in PBS and PBS with elastase K or α-chymotrypsin. As shown in Figure 4a, there was a mass loss of over 70% and 67% hydrogel in the elastase K and α-chymotrypsin groups on day 15, respectively—considerably higher than 20% in PBS. The results could be explained by the absence of elastase K or α-chymotrypsin, where the mass loss of gels was merely attributed to the surface erosion of the hydrogels. However, with elastase K or α-chymotrypsin, the polypeptide chains of the hydrogels were also fast degrading, which sped up the loss of gels.

The degradation process of hydrogels in vivo was monitored for 28 days, and images were taken at 10 min, 7, 14, 21, and 28 days after hydrogel treatment. At 4 °C, 500.0 μL of mPEG_45_−PLAla_30_ solution (5.0 wt. %) was subcutaneously injected into Sprague−Dawley (SD) rats through 21-gauge syringe needles. In Figure 4b, the solution rapidly turned into gel in 10 min after injection. Seven days later, only a small portion of the hydrogel was degraded. On day 14, the size of the hydrogel shrank to less than 50%, while on day 28, the hydrogel was completely degraded. This degradation rate of hydrogel was suitable for application in biomedical fields requiring long-term treatment, such as tissue repair. As shown in Figure 4c, at different time intervals, the tissue surrounding the gel was surgically separated and processed by H&E staining to examine the condition of tissue damage. Basically, no histological damage or immune response was found at any time point.

### 2.6. Safety Evaluation

**Figure 5:**
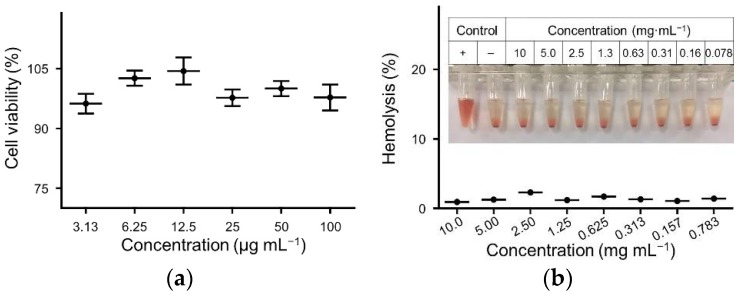
Biological compatibility of mPEG_45_−PLAla_30_. (**a**) In vitro cytotoxicity of the mPEG_45_−PLAla_30_ toward L929 cells. Data were presented as mean ± SD (*n* = 5); (**b**) Hemolysis experiments of mPEG_45_−PLAla_30_. Data are presented as mean ± SD (*n* = 3).

The cytotoxicity of hydrogel mPEG_45_−PLAla_30_ in vitro was evaluated by methyl thiazolyl tetrazolium (MTT) assay and hemolysis test. In Figure 5a, L929 cells treated with the highest concentration of mPEG_45_−PLAla_30_ (100.0 ìg mL^−1^) for 24 h retained almost 100% viability, confirming its excellent biocompatibility. Moreover, the effect of different concentrations of gel on hemolysis was also investigated. In this part, hemolysis was measured spectroscopically according to previously reported methods [39]. As shown in Figure 5b, no hemolysis was detected in blood samples treated with all test concentrations of mPEG_45_−PLAla_30_, which further confirmed the outstanding biocompatibility. Overall, the findings above revealed that the hydrogel showed little toxicity, both in vitro and in vivo. Therefore, it is a promising material for usage in clinical practice.

## 3. Materials and Methods

### 3.1. Materials

mPEG−OH, number-average molecular weight (*Mn*) = 2000 g mol^−1^, was purchased from Sigma-Aldrich (St. Louis, MO, USA). The mPEG_45_−NH_2_ was synthesized conforming to the previously reported protocol in our work [40]. THF and toluene were refluxed with sodium and distilled under nitrogen before usage. *N*,*N*-Dimethylformamide (DMF) was stored over calcium hydride (CaH_2_) and purified by vacuum distillation. All the other reagents and solvents were bought from Sinopharm Chemical Reagent Co. Ltd., Beijing, China, and used as obtained.

### 3.2. Phase Diagram

The sol–gel transition behavior of the copolymers in PBS (pH 7.4) was determined by inverting test method with a temperature increment of 2 °C per step. Samples with concentrations ranging from 3.0−7.0 wt. % were dissolved in PBS and stirred at 0 °C for 12 h. The copolymer solution (0.2 mL) was introduced into the test tube with an inner diameter of 10.0 mm. The sol–gel transition temperature was recorded if no flow was observed within 30 s after inverting the test tube. Each data point was the average of three measurements.

### 3.3. Dynamic Mechanical Analysis

Rheological experiments were performed on a US 302 Rheometer (Anton Paar, Graz, Austria). The copolymer solution was placed between parallel plates of 25.0 mm in diameter with a gap of 0.5 mm. To prevent the evaporation of water, the outer edge of the sandwiched sample was sealed by a thin layer of silicon oil. The data were collected under a controlled strain γ of 1% and a frequency of 1 rad s^−1^. The heating rate was 1 °C min^−1^.

### 3.4. In Vitro Gel Degradation

For this, 0.5 mL of mPEG_45_−PLAla_30_ hydrogel was incubated in PBS (5.0 wt. %) in vials (diameter = 16.0 mm) at 37 °C for 10 min. PBS solutions (pH 7.4) containing 0.2 mg mL^−1^ elastase K or 0.2 mg mL^−1^ α-chymotrypsin were used as degradation media, and hydrogels incubated in PBS were only used as a control. Different solutions (2.0 mL) were added to the top of the gels at 37 °C and the entire medium was changed daily. The weight of the remaining gel was measured daily.

### 3.5. In Vivo Gel Degradation

SD rats (about 180.0 g, provided by Beijing Vital River Laboratory Animal Technology Co., Ltd., Beijing, China) were used for the detection of gel degradation in vivo. Rats were anesthetized by inhalation of ether before 0.5 mL of mPEG_45_−PLAla_30_ PBS solutions (5.0 wt. %) were injected into the dorsal subcutaneous area of the rats using a 21-gauge needle. The rats were sacrificed on day 7, day 14, day 21, and day 28, respectively, to monitor the degradation behavior of the gel. The experiments on animals were carried out according to the guide for the care and use of laboratory animals, provided by Jilin University, Changchun, China, and the procedure was approved by the local Animal Ethics Committee.

### 3.6. Cytotoxicity Measurement

The relative cytotoxicity was assessed by MTT viability assay against L929 mouse fibroblasts cells. L929 cells were cultured in complete Dulbecco’s modified Eagle’s medium (DMEM) supplemented with 10.0% (*v*/*v*) FBS, penicillin (50.0 IU mL^−1^), and streptomycin (50.0 IU mL^−1^) at 37 °C in a 5.0% (*v*/*v*) carbon dioxide atmosphere. L929 cells with a density of 6000 cells per well were planted in 96-well plates in 180.0 μL of DMEM. After incubation for 24 h, 20.0 μL of copolymer solutions at different concentrations (31.3–1000.0 μg mL^−1^) were added. L929 cells were incubated with copolymers for another 24 h before 20.0 ìL of PBS solution containing MTT (0.05 mg mL^−1^) was added and incubated for a further 4 h. Then, the media was replaced with 160.0 μL of dimethyl sulfoxide (DMSO). The absorbance of the solution was measured on a Bio-Rad 680 microplate reader (Hercules, CA, USA) at 490 nm. Cell viability (%) was calculated according to the following Equation (1).

Measurements were done in five replicates.

(1) Cell viability (%) = A_sample_/A_control_ × 100,

where A_sample_ and A_control_ denote the absorbances of sample and control, respectively.

## 4. Conclusions

In this work, thermosensitive hydrogels mPEG_45_−PLAla with three different DP (14, 22, and 30) were synthesized by ROP of l-Ala NCA monomer initiated by mPEG_45_−NH_2_. The effect of different DP on the solution−gel transition was investigated. It is worth noting that mPEG_45_−PLAla_30_ had a lower sol–gel transition temperature than mPEG_45_−PLAla_22_, attributed to the longer hydrophobic segment. In addition, mPEG−PLAla hydrogel had good stability and high mechanical strength after gelation. Moreover, mPEG−PLAla hydrogel did not cause any tissue damage, inflammatory reaction, or hemolysis reaction during degradation, confirming its good biocompatibility. In summary, the mPEG−PLAla hydrogel designed in our study had improved biocompatibility, appropriate degradation, and gelation ability. Therefore, the polypeptide hydrogels showed promise for application in tissue repair and regeneration, cell 3D culture, and treatment of cancer. Moreover, by loading functional drugs, the hydrogels can also be used for postoperative recovery and anti-infection. Based on the above findings, polypeptide thermosensitive hydrogels have broad prospects in the biomedical field.

## Supplementary Materials

The following are available online.
