# Supplementary material for: Effect of Hydrophobic Polypeptide Length on Performances of Thermo-Sensitive Hydrogels
Source: Molecules. 2018 Apr 26;23(5):1017. doi: 10.3390/molecules23051017 (PMC6102579; doi:10.3390/molecules23051017)
Supplement: Supplementary file 1 [file molecules-23-01017-s001.pdf]

molecules

Supplementary Materials

Effect of Hydrophobic Polypeptide Length on Performances of Thermo-Sensitive Hydrogels

Jiandong Han [1,2], Xingyu Zhao [2], Weiguo Xu [2], Wei Wang [1,*], Yuping Han [2,3,*] and Xiangru Feng [2,*]

[1] Department of Chemistry, Changchun University of Science and Technology, Changchun 130022, P. R. China; jdhan@ciac.ac.cn (H.J.)

[2] Key Laboratory of Polymer Ecomaterials, Changchun Institute of Applied Chemistry, Chinese Academy of Sciences, Changchun 130022, P. R. China; star20012002@163.com (Z.X.); wgxu@ciac.ac.cn (X.W.)

[3] Department of Urology, China-Japan Union Hospital of Jilin University, Changchun 130033, P. R. China

* Correspondence: weiwanglg@163.com (W.W.); hyp181818@126.com (H.Y.); xrfeng@ciac.ac.cn (F.X.)

Received: date; Accepted: date; Published: date

Materials and Methods

Characterization

^{1}H NMR spectra of $mPEG_{45}$–PLAla in deuterated trifluoroacetic acid (CF_3COOD) were conducted on a Bruker AV 300 NMR spectrometer. ^{13}C NMR spectral changes of $mPEG_{45}$–$PlAla_{30}$ (5.0 wt.% in D_2O) were investigated as a function of temperature between 20 and 60 °C on a Bruker AV 400 NMR spectrometer. The solution temperature was equilibrated for 20 min before measurement. The ellipticity of polymer aqueous solution (0.05 mg mL^{-1}) was obtained on a JASCO J-810 spectrometer as a function of temperature between 10 and 50 °C. DLS measurements were determined on a WyattQELS instrument with a vertically polarized He–Ne laser (DAWN EOS, Wyatt Technology) and 90° collected optics. The sample was prepared in aqueous solution at the concentration of 5.0 μg mL^{-1}. The solution was filtered through a 0.45 μm Millipore filter before measurements. FT-IR spectra were recorded on a Bio-Rad Win-IR instrument using potassium bromide method.

Synthesis of L-Ala NCA

L-alanine (20.0 g, 0.224 mol) and triphosgene (53.4 g, 0.180 mol) were suspended in 400.0 mL of dry THF bubbled with nitrogen flux in a flame-dried three-neck flask. The mixture was stirred at 60 °C for 2 h before further bubbling with nitrogen flux for 30 min. After that, the solution was precipitated in 1000.0 mL of *n*-hexane and stored at −20 °C. The supernatant was removed, and the residues were collected and dissolved in 200.0 mL of ethyl acetate, prior to two washings with 100.0 mL of ice-cold water and one washing with 100.0 mL of 0.5% $NaHCO_3$ ice-cold aqueous solution. The organic phase was then dried over anhydrous $MgSO_4$ and evaporated to obtain 15.5 g of L-Ala NCA. The yield of L-Ala NCA was 77.5%.

Synthesis of $mPEG_{45}$*–PLAla*

The $mPEG_{45}$-polypeptide copolymers were synthesized through the ROP of L-Ala NCA using $mPEG_{45}$-NH_2 as macroinitiator. The following was a typical procedure for the preparation of $mPEG_{45}$–$PLAla_{30}$: $mPEG_{45}$-NH_2 (2.0 g, 0.001 mol) was dissolved in toluene (150.0 mL) and residual water in the solution was removed by azeotropic distillation. Anhydrous DMF (100.0 mL) and L-Ala NCA (3.2 g, 0.028 mol) were then added to the flask. The reaction mixture was stirred at 25 °C for three days under a dry nitrogen atmosphere. Then the copolymer was purified by precipitation in glacial diethyl ether, followed by filtration. The resulting product was dissolved in DMF and dialyzed in a dialysis bag (molecular weight cut-off (MWCO) = 3500 Da) for three days. The water was changed every six hours to remove the DMF. Then the final product was obtained by lyophilization. The yield of $mPEG_{45}$–$PLAla_{30}$ was 73.4%. Similarly, $mPEG_{45}$–$PLAla_{22}$ and $mPEG_{45}$–$PLAla_{14}$ were synthesized according to the abovementioned protocol by changing the feed amounts of L-Ala NCA, which were 2.4 g (0.021 mol) and 1.6 g (0.014 mol), respectively. The yields of $mPEG_{45}$–$PLAla_{22}$ and $mPEG_{45}$–$PLAla_{14}$ were 61.1% and 57.7%, respectively.

Hydrogel Internal Morphology

The internal morphology of the hydrogel was observed using a field emission scanning electron microscope (ESEM, Micrion FEI PHILIPS). After the hydrogel was formed, it was rapidly frozen with liquid nitrogen and lyophilized to acquire a lyophilized gel sample. The lyophilized samples were evenly sprayed with gold after brittle fracture, and the gel internal morphology and pore size were observed under ESEM.

Histological Analyses

SD rats were sacrificed after the *in vivo* degradation experiment. The hydrogels near the skin were removed and fixed in 4.0% (w/v) PBS-buffered paraformaldehyde overnight and then embedded in paraffin. Paraffin-embedded tissues were sectioned into approximately 5.0 μm slices

and stained with H&E. The histological changes were detected by a microscope (Nikon Eclipse *Ti*, Optical Apparatus Co., Ardmore, PA).

Hemolysis Tests

The hemocompatibility level of $mPEG_{45}$–$PLAla_{30}$ was determined according to established criteria, ISO 10993-4. Briefly, the fresh rabbit blood was purchased from the Laboratory Animal Center of Jilin University, which was obtained from the heart of a live rabbit. Subsequently, it was diluted by normal saline (NS), and then the red blood cells (RBCs) were isolated from plasma by centrifugation at 2500 rpm for 15 min. After careful washing, the suspension of RBCs at a final concentration of 2.0% (v/v) was added to $mPEG_{45}$–$PLAla_{30}$ solution with varied concentrations, mixed by vortex, and then incubated at 37 °C in a thermostatic water bath for 2 h. NS and Triton X-100 (1×10^4 μg mL^{-1}, a surfactant known to lyse RBCs) were used as negative and positive controls, respectively. Then, RBCs were centrifuged at 3000 rpm for 10 min, and then 100.0 μL of the supernatant of each sample was transferred to a 96-well plate. The free hemoglobin in the supernatant was measured with a Bio-Rad 680 microplate reader at 540 nm. The hemolysis ratio of RBCs was calculated using Equation (2).

$$\text{Hemolytic ratio (\%)} = (A_{sample} - A_{negative\ control}) / (A_{positive\ control} - A_{negative\ control}) \times 100 \quad (1)$$

Where, A_{sample}, $A_{negative\ control}$, and $A_{positive\ control}$ were denoted as the absorbencies of sample, and negative and positive controls, respectively.
